# Erysipelas-Like Carcinoma of the Parotid Gland With Cutaneous Metastasis to the Neck Requiring Tracheostomy: A Case Report

**DOI:** 10.7759/cureus.66136

**Published:** 2024-08-04

**Authors:** Natsuko Saito-Sasaki, Tomoko Oda, Etsuko Okada, Yu Sawada

**Affiliations:** 1 Dermatology, University of Occupational and Environmental Health, Kitakyushu, JPN

**Keywords:** parotid gland, carcinoma ex pleomorphic adenoma, erysipelas-like carcinoma, tracheostomy, metastasis

## Abstract

We present the case of a 71-year-old man who, after undergoing postoperative radiotherapy for epithelial carcinoma, developed progressively enlarging erythema. Initially, the condition resembled radiation dermatitis or erysipelas, and topical steroids and antibacterial agents were administered without success. A biopsy was performed for further evaluation, revealing a cutaneous invasion of parotid carcinoma. The lesion continued to enlarge, leading to dysphagia and ultimately necessitating a tracheostomy.

## Introduction

Radiotherapy is frequently employed to treat head and neck cancers, but high doses of radiation can often lead to skin damage. Risk factors for this adverse effect include concurrent chemotherapy, advanced TNM stage (stage IV), and older age. Damage to the skin and mucous membranes can disrupt treatment protocols, require hospitalization, and significantly impair quality of life [1].

The parotid gland, a salivary gland situated in front of and below the ear, is vulnerable to various malignant tumors. These include mucoepidermoid carcinoma, adenoid cystic carcinoma, and adenoid carcinoma, among others. More than 20 distinct histological types of tumors have been identified in this gland [2,3].

Carcinoma ex pleomorphic adenoma is a high-grade malignant ductal tumor that frequently leads to local recurrence or distant metastasis, even following postoperative chemoradiation therapy [4]. Surgical resection of parotid carcinoma can be classified into several techniques: partial parotidectomy, parotid lobectomy (both shallow and deep lobes), total parotidectomy, and total parotidectomy with expansion. Radiation therapy is recommended as an adjuvant treatment in high-grade tumors and in cases of incomplete resection [5].

Here, we report a case of erysipelas-like carcinoma of the parotid gland that led to neck strangulation and necessitated a tracheostomy.

## Case presentation

A 71-year-old male presented with a right mandibular mass that had been present for approximately five years. After metastasis to the cervical lymph nodes was diagnosed via fine needle aspiration, he underwent primary tumor resection and cervical lymph node dissection. The diagnosis was parotid carcinoma ex pleomorphic adenoma, staged pathologically as T2N2bM0 (stage IVA). A PET-CT scan was not conducted for this patient.

**Figure 1:**
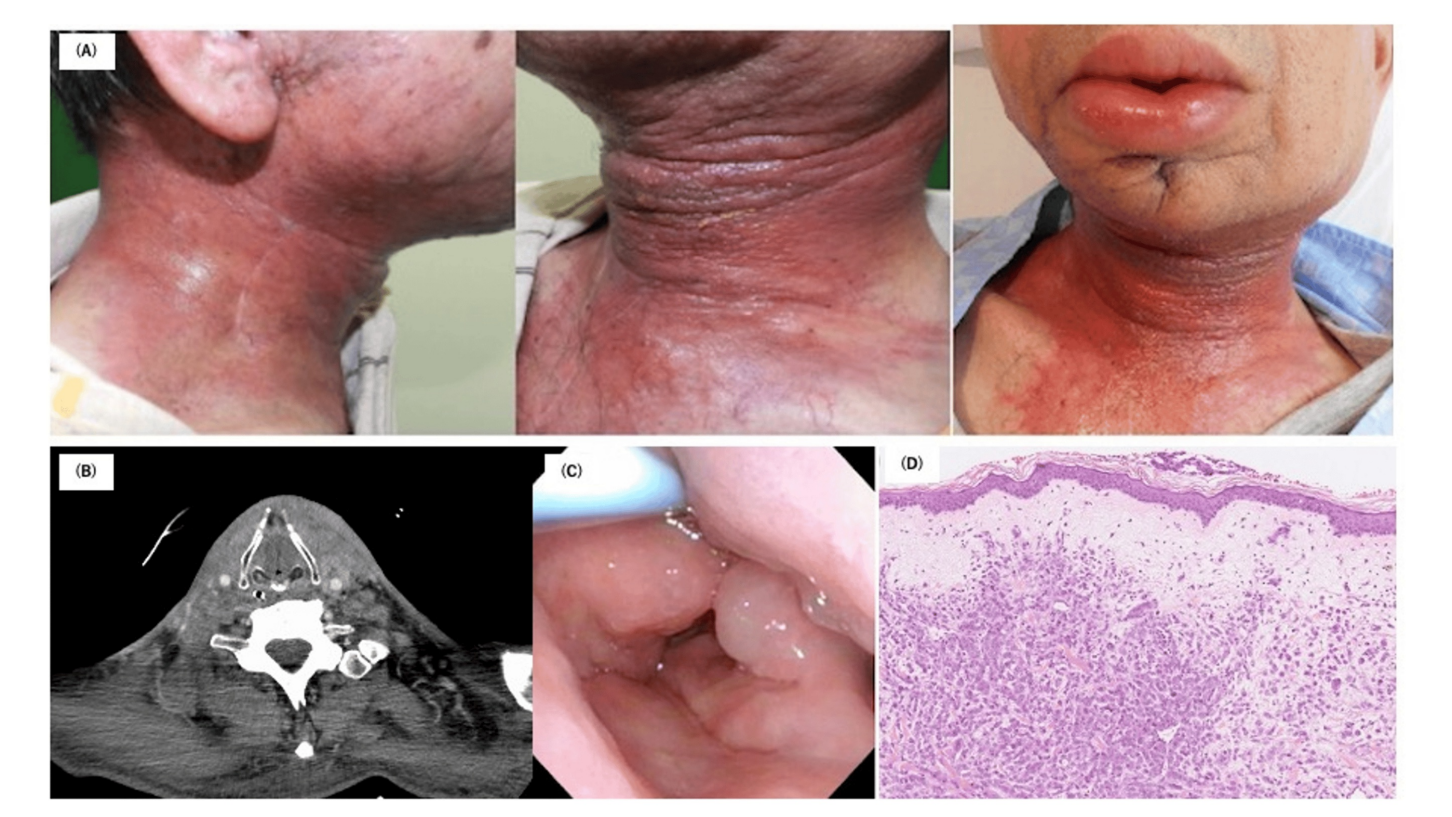
Clinical manifestation, CT imaging, and histological examination (A) Initial clinical manifestation of the tumor’s cutaneous development and subsequent invasion across the entire neck and face. (B) CT imaging. (C) Laryngeal edema prior to tracheostomy. (D) Histological examination of the skin showing diffuse or irregular proliferation of atypical cuboidal or polygonal epithelial cells, occasionally with intracytoplasmic lumens, arranged in cords or small nests within the dermis. Immunohistochemically, the carcinoma cells are positive for AE1/AE3, CAM5.2, and HER2, and negative for androgen receptors, GCDFP-15, alpha-SMA, and GATA-3.

At the early stage of the disease, the tumor was localized with no evidence of local invasion. Consequently, he received postoperative radiotherapy of 70 Gy in 35 fractions to the right side of the neck and jaw. One month after radiotherapy, erythema developed gradually and was initially slightly improved with treatment. However, a swollen erythema resembling radiation dermatitis was observed on the contralateral left neck. Topical steroids and antibiotics were administered, but the erythema proved refractory, rapidly progressing to involve the entire neck, cheeks, and lips (Figure 1A).

The patient was referred to our department for an evaluation of the rash. A CT scan revealed swelling of the subcutaneous tissue without obvious tumor formation (Figure 1B). Due to laryngeal edema, a tracheotomy was performed (Figure 1C). A skin biopsy from the neck showed a diffuse or haphazard proliferation of atypical cuboidal or polygonal epithelial cells, occasionally with intracytoplasmic lumens, arranged in cords or small nests, involving local subcutaneous invasion of the primary carcinoma (Figure 1D). Immunohistochemically, the carcinoma cells were positively reactive to AE1/AE3, CAM5.2, and HER2, while androgen receptors, GCDFP-15, alpha-SMA, and GATA-3, were negative.

Chemotherapy was not administered as the patient’s general condition deteriorated rapidly. Within a week of the diagnosis, he experienced difficulty swallowing, and a tracheotomy was added. Unfortunately, due to his poor general condition, he developed pneumonia and passed away a month later.

## Discussion

This is the first case study describing erysipelas-like skin eruptions associated with parotid gland cancer. Clinicians should be aware that such skin eruptions may indicate cutaneous metastases involving the neck, potentially necessitating tracheostomy treatment.

Erysipelatoid carcinoma presents as papuloerythematous, infiltrative-edematous, and slightly indurated lesions that resemble acute infectious processes, such as erysipelas and radiation-induced skin disorders [6-9]. While the exact mechanism remains unclear, two possible explanations for the subcutaneous tumor invasion are proposed.

The first possibility is that the radiation treatment area might have been insufficient to control tumor growth. Lymph node metastasis and neck dissection could have led to new lymphatic pathways, facilitating the aggressive migration of the tumor to the contralateral side [10].

The second possibility is that radiation itself may activate local tumor cell migration. Although radiation generally exerts a cytotoxic effect on tumor cells, some studies suggest that it can enhance the migratory capacity of tumor cells. In this patient, the initial clinical manifestation of skin invasion occurred at the site opposite the primary tumor and subsequently spread across the entire neck and face.

While a fluctuation in lymphatic flow or the microinvasive behavior of the tumor might contribute to this unusual skin development, these findings highlight the need for caution when using radiotherapy for malignancies characterized by aggressive local invasion.

## Conclusions

In cases of head and neck cancer, topical steroids are sometimes used to treat suspected dermatitis following neck radiation therapy. However, if the condition is refractory and continues to progress, a biopsy should be conducted promptly to rule out a cancer invasion. Dermatologists should be aware that erysipelas-like invasive carcinoma of the neck can lead to life-threatening complications such as asphyxia and should pursue thorough testing as needed.
